# Retrospect on the Ground Deformation Process and Potential Triggering Mechanism of the Traditional Steel Production Base in Laiwu with ALOS PALSAR and Sentinel-1 SAR Sensors

**DOI:** 10.3390/s24154872

**Published:** 2024-07-26

**Authors:** Chao Ding, Guangcai Feng, Lu Zhang, Wenxin Wang

**Affiliations:** 1 School of Civil Engineering and Geomatics, Shandong University of Technology, Zibo 255049, China; 2 State Key Laboratory of Information Engineering in Surveying, Mapping and Remote Sensing, Wuhan University, Wuhan 430079, China; luzhang@whu.edu.cn; 3 School of Geosciences and Info-Physics, Central South University, Changsha 410083, China; fredgps@csu.edu.cn (G.F.); wangwx@csu.edu.cn (W.W.)

**Keywords:** SBAS-InSAR, ground deformation, underground ore mining, tailing pond, triggering mechanism, risk evaluation

## Abstract

The realization of a harmonious relationship between the natural environment and economic development has always been the unremitting pursuit of traditional mineral resource-based cities. With rich reserves of iron and coal ore resources, Laiwu has become an important steel production base in Shandong Province in China, after several decades of industrial development. However, some serious environmental problems have occurred with the quick development of local steel industries, with ground subsidence and consequent secondary disasters as the most representative ones. To better evaluate possible ground collapse risk, comprehensive approaches incorporating the common deformation monitoring with small-baseline subset (SBAS)-synthetic aperture radar interferometry (InSAR) technique, environmental factors analysis, and risk evaluation are designed here with ALOS PALSAR and Sentinel-1 SAR observations. A retrospect on the ground deformation process indicates that ground deformation has largely decreased by around 51.57% in area but increased on average by around −5.4 mm/year in magnitude over the observation period of Sentinel-1 (30 July 2015 to 22 August 2022), compared to that of ALOS PALSAR (17 January 2007 to 28 October 2010). To better reveal the potential triggering mechanism, environmental factors are also utilized and conjointly analyzed with the ground deformation time series. These analysis results indicate that the ground deformation signals are highly correlated with human industrial activities, such underground mining, and the operation of manual infrastructures (landfill, tailing pond, and so on). In addition, the evaluation demonstrates that the area with potential collapse risk (levels of medium, high, and extremely high) occupies around 8.19 km^2^, approximately 0.86% of the whole study region. This study sheds a bright light on the safety guarantee for the industrial operation and the ecologically friendly urban development of traditional steel production industrial cities in China.

## 1. Introduction

With iron and coal ores as two dominant minerals, in the last few decades, Laiwu has developed into one of the dominant production areas for steel products in Shandong Province in China. Here, for the simplicity of expression, “Laiwu” indicates the original prefecture-level city composed of Laiwu District and Gangcheng District, which were merged into the provincial capital of Shandong Province in January 2019. The iron ore deposits in Laiwu have proven basic reserves, accounting for approximately one fifth of the reserves in the whole Shandong Province [1]. Moreover, as an important coal production base, there are also massive coal mining activities in Laiwu coal fields to supply ample coal for local steel companies. Possessing a complete industrial system for mining and processing iron and coal ores, Laiwu has become one of the largest steel producers of Shandong Province. However, except for bringing about the quick development of the local economy and society, the long-term underground mining activities excavated out lots of ore resources and left a lot of underground goaf.

Furthermore, due to the specific geological and underground hydrological conditions, karst underground water systems are widely distributed in the iron and coal ore mining region. Mine drainage will first be performed to extract out a large amount of underground karst water, and facilitate the occurrence of a “skylight” [2,3]. Notably, a skylight indicates a phenomenon with serious mass migration and overlying material subsidence that occurred between a Quaternary stratum and an Ordovician stratum [4]. The ore mining and mine drainage directly destroy the dynamic equilibrium of local geological conditions and possibly trigger the occurrence of karst collapse events. The Laiwu Basin is one of the areas with the highest concentration of karst collapse in Shandong Province [5,6,7], coupled with a large population and well-developed economy. However, the ground deformation is the quantity that directly reflects the degree of ground subsidence triggered by the fluctuations of karst water levels [8,9,10]. Thus, to prevent potential karst collapse and the secondary geological disasters in Laiwu, a risk evaluation based on the ground deformation signals may have significance in guiding urban development and the effective utilization of mineral resources. However, these existing studies are mostly focused on historical ground subsidence and collapse events before 2015 [6,8,11], which have lost the time sensitivities for analyzing the current ground deformation and possible ground collapse risks. Deriving the ground deformation process of recent years can be helpful for updating knowledge of the local deformation status of the traditional steel production base in Laiwu, and further predicting possible collapse risks in the future.

To better demonstrate the ground deformation history and the internal triggering mechanism, this study is performed with the following sections. Firstly, information on the study area, remote sensing, and auxiliary datasets are introduced in Section 2.1. Then, the methodological procedures of SBAS-InSAR, deformation analysis, and risk evaluation are described, given in Section 2.2. Subsequently, the ground deformation results are displayed in Section 3. Afterwards, the spatiotemporal deformation characteristics, the potential triggering mechanism, and the collapse risks are qualitatively and quantitatively discussed in Section 4.

## 2. Materials and Methods

### 2.1. Study Area and Datasets

#### 2.1.1. Study Area

**Figure 1:**
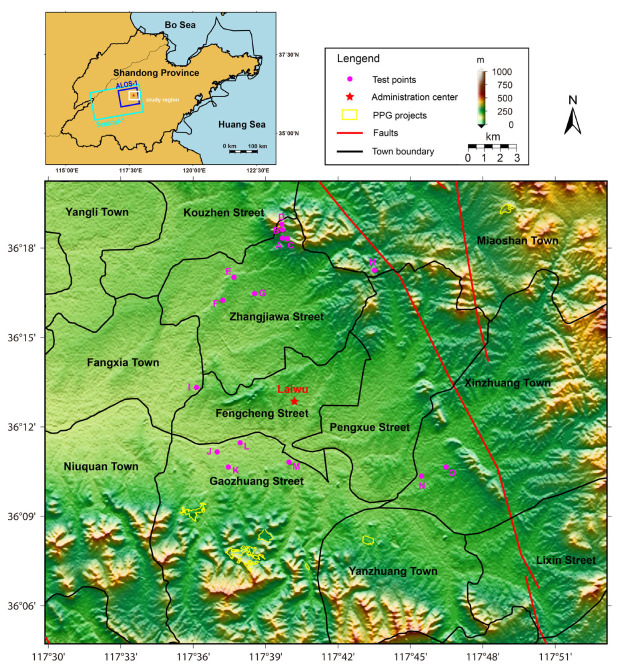
The geographical background of the study area in Laiwu, in which the image coverages for ALOS PALSAR and Sentinel-1 are contoured with blue and cyan polylines, respectively. The background image of this study region is the topographic map. Notably, indicated by the magenta dots, 15 test points coded from A to O are located at Yujiaquan tailing pond (A, B, C, D), the iron ore mining region (E, F, G, H, I), the banksides of the Dawen River (J, K, L), and the coal mining region (M, N, O).

The study region is situated in the Xintai Coalfield and mainly belongs to the geomorphology of an intermountain plain. Located at the Taiyi Mountains of central Shandong Province as shown in Figure 1, Laiwu has a geological structure controlled by the latitudinal structure of Central Shandong and the circinate structure of Western Shandong. As a semicircular basin protruding to the north, Laiwu is surrounded by the northern, eastern, and southern mountains, with the Dawen River running through the middle of the basin from east to west. Additionally, Laiwu has a warm, temperate continental monsoon climate, with high precipitation and high temperature in the summer months, and infrequent precipitation and low temperature in the winter months.

Famous for its well-developed steel industry supply chain, Laiwu is a traditional mining city with advantageous minerals including coal, iron ore, limestone, dolomite, and other mineral resources. The exploitation of mineral resources has promoted local economic development, but also damaged the ecological environment. For example, driven by daily mining and mineral separating activities, ground deformation is the dominant factor in triggering the possible karst collapse and the dam break of tailing ponds. To better understand the structure of the local steel production base, specific descriptions of the ore mining and tailing pond projects can be found in Section 2.1.2 and Section 2.1.3.

#### 2.1.2. Traditional Mining Operation of Iron and Coal Ores

Possessing an explored reserve of approximately 400 million tons, Laiwu is an important base of high-grade iron ore deposit in China [12]. Located at the major coal accumulation period of the Carboniferous–Permian [13], the Laiwu coalfield is also one of the important coal-forming areas with more than 20 coal layers [14]. Up to now, there are proven coal reserves of around 300 million tons in Laiwu [15]. The structures of Laiwu’s faults and folds are complex due to multi-stage geological tectonic movement. At present, the local coal mining enterprises mainly incorporate three state-owned coal mines of the Xinwen Mining Group: Ezhuang, Huatai, and Panxi.

Due to the widely distributed karst landforms, the underground karst water resources in the ore mining region are very rich. Before retrieving high-valued iron and coal ores, mine drainage must be performed and it directly triggers a decrease in the underground water level. Subsequently, the karst caves possibly facilitate the occurrence of karst collapse events. Triggered by the widely distributed ore mining operations and fragile geological conditions, massive ground deformation indications are found in the study area. The expansion of the ground subsidence area seriously restricts local social and economic development.

#### 2.1.3. Tailing Pond Projects for Screening Iron Ore

As an indispensable engineering facility for mining enterprises, the tailing pond is commonly used to stack tailings and industrial waste residue generated from production operations [16]. However, after a long period of discharge and accumulation, the tailing pond generally becomes a hazard source of artificial debris flow with a high potential energy [17]. Long-term and large-scale human engineering activities will inevitably lead to the ground surface deformation of tailing ponds, resulting in accidents such as dam collapse. Once the tailing pond fails, it will not only seriously threaten the safety of downstream residents but also damage the buildings and facilities. In recent years, remarkable results have been achieved in the special rectification and comprehensive treatment of tailing ponds, but production safety accidents still occur sporadically. Improving the safety monitoring efficiency of tailing ponds, identifying hazards, and extracting disaster information in a timely manner can effectively resolve the safety risks, which is of great significance for the safety management of tailing ponds.

In total, there are three tailing ponds for screening iron ore in Laiwu, which are a Class-five tailing pond belonging to the Chuiyang Iron Company, a Yaojialing Class-five overhand tailing pond belonging to the Kanghua Mining Company, and a Yujiaquan Class-three overhead tailing pond belonging to the Luzhong Mining Company [18], of which, the Yujiaquan Class-three tailing pond is the largest overhead reservoir of Shandong Province [19,20]. As an indispensable engineering facility for mining enterprises in mineral processing, this tailings pond has formed a large man-made hazard source of debris flow with high potential energy, after a long period of discharge and accumulation. Many human settlements, traffic road networks, and industrial infrastructures are distributed downstream of the Yujiaquan tailing pond. Also, the two large water reservoirs of Dazhi and Qingyang are located close to the north and south sides of the Yujiaquan tailing pond, seriously polluted by the tailings when the dam collapse events occurred. Frequent monitoring on the ground deformation of this dam is, thus, very crucial for guaranteeing the safe operation of the Yujiaquan tailing pond and also the safety of downstream infrastructures in the neighborhood.

#### 2.1.4. Remote Sensing Datasets

Here, ALOS PALSAR and Sentinel-1 SAR datasets are conjointly utilized for monitoring the ground deformation process in Laiwu. In total, 22 ALOS PALSAR images and 176 Sentinel-1 images over different observation periods are retrieved. The specific sensor parameters and acquisition details of these two sensors can be found in Table 1. Additionally, to provide a clearer optical presentation and validation of the temporal changes of the ground surface, the multi-temporal optical dataset of Sentinel-2 with a 10 m spatial resolution is also utilized in this study.

#### 2.1.5. Auxiliary Datasets

To better demonstrate the triggering mechanism of potential environment factors on ground deformation and collapse, the precipitation dataset is freely downloaded from the NASA website [21]. In addition, the observatory records (magnitude, date, geological location, depth, etc.) of local earthquake events are freely downloaded from the Chinese Earthquake Networks Center (CENC) [22]. Additionally, to achieve a more comprehensive knowledge of the social and economic factors, the dominant road networks and industrial infrastructure are fully retrieved from the Chinese National Geographical Information Public Service Platform of Tianditu System [23]. Moreover, the buildings’ spatial distribution and the average building heights with 10 m resolution of the study region are used here [24].

### 2.2. Methods

**Figure 2:**
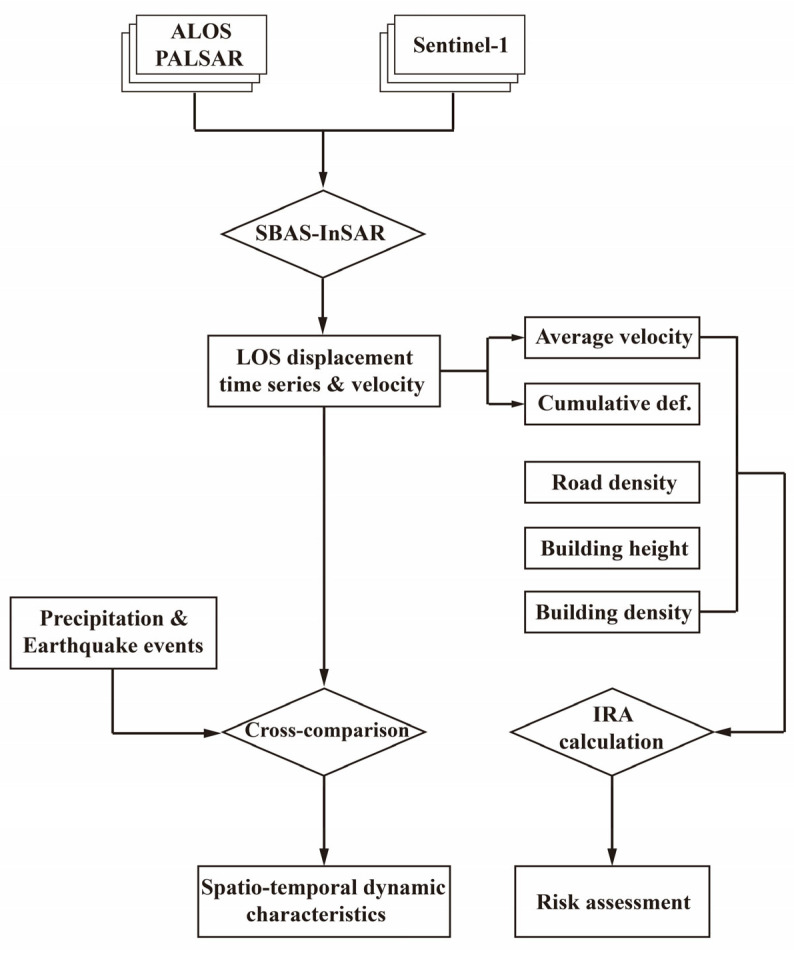
Diagram of the methodology utilized for retrieving the ground deformation process, analyzing the potential triggering mechanisms, and evaluating the possible subsidence risks.

In this study, the small-baseline subsets InSAR (SBAS-InSAR) technique [25,26] is used to derive the ground deformation time series of the study area. Then, the deformation time series and environmental factors are utilized for conjointly analyzing the potential influential mechanism. Subsequently, a risk evaluation is performed to evaluate the safety operation of local infrastructures in the iron ore and coal-producing area. The detailed processing procedures for the methodology are presented in Figure 2.

#### 2.2.1. SBAS-InSAR Processing

Assuming there are totally *N* + 1 images for the ALOS PALSAR or Sentinel-1 SAR sensor, *M* pairs of InSAR light-of-sight (LOS) measurements $\delta d_{j}(j=1,\ldots ,\;M)$ will be generated after using the “Small-baseline Subset” pairing strategy. If the average displacement velocities for two arbitrary adjacent time separations $\left(t_{i}-t_{i-1}\right)\;(i=1,2,\ldots ,\;N)$ are denoted by $v_{k}$, the cumulative deformation $D_{i}$ can be calculated from Equation (1).

(1) $$D_{i}=\left\{\begin{array}{c} 0,\;\;\;\;i=0\\ \sum_{k=1}^{i}v_{k}\left(t_{k}-t_{k-1}\right),\;\;\;i=1,\;\;\;2,\;\ldots ,\;N \end{array}\right.$$

Each InSAR measurement is generated by two master-slave SAR single look complex (SLC) image pairs acquired at $t_{m_{j}}$ and $t_{s_{j}}$, in which $t_{m_{j}}>t_{s_{j}}(j=1,2,\ldots ,\;M)$, then $d_{j}(j=1,2,\ldots ,\;M)$ can be derived from Equation (2).

(2) $$d_{j}=D_{m_{j}}-D_{s_{j}}=\sum_{k=s_{j}}^{m_{j}}v_{k}\left(t_{k}-t_{k-1}\right),\;\;\;j=1,\;2,\;\ldots ,\;M$$

With $v_{k}$ as the physical parameters to be resolved, Equation (2) can be subsequently simplified into Equation (3). Specifically, assuming the coefficient matrix B is nonsingular, the velocity parameter v can be resolved following the least-squared inversion rationale as displayed in Equation (3), in which P denotes the weighting matrix decided by the coherence of each InSAR measurement. However, if the coefficient matrix *B* is rank-deficient, a singular value decomposition approach is applied for *B* to resolve the velocity parameter v [25,26]:

(3) $$v={\left(B^{T}PB\right)}^{-1}B^{T}Pd$$

In this study, the module of Interferometry, Diff. Interferometry and Geocoding (ISP/DIFF&GEO) embedded in GAMMA Version Linux64 16.04 software is utilized for the aforementioned SBAS-InSAR processing procedures. Specifically, the number of looks is configured as 3 (range) × 8 (azimuth) for ALOS PALSAR and 5 (range) × 1 (azimuth) for Sentinel-1. In the specific SBAS-InSAR processing, the spatial and temporal baseline thresholds are configured as 75~1200 m/45~900 days for ALOS PALSAR and 0~200 m/12~180 days for Sentinel-1.

#### 2.2.2. Deformation Post-Processing and Risk Evaluation

After retrieving the LOS-oriented deformation time series and velocity, these deformation results are further analyzed to better demonstrate the spatiotemporal evolution patterns. Specifically, the deformation regions for different sensors are extracted through a 3-sigma iterative filter [27]. Then, the potential influential factors incorporating precipitation, earthquake events, and human activities are utilized for the conjoint analysis on the ground deformation process and the internal triggering mechanism.

In addition, to better evaluate the potential collapse risks of the ground deformation facing Laiwu, the revised method of Ding et al., 2024 [28] is proposed in this subsection. For the reason that the ground deformation region mainly occurs in the farmland and industrial region without extensive building cover, the spatial linear density of traffic road networks is utilized in two factors evaluating the ground stability and the risk levels. Specifically, the Index of Risk Evaluation (IRE) for each pixel is designed by combining the average deformation velocity $\bar{v}$, the cumulative deformation magnitude *D*, the spatial line density of traffic road networks $\rho_{r}$, the spatial point density of local buildings $\rho_{b}$, and the average height of local buildings ${\bar{h}}_{b}$, as presented in Equation (4). Notably, the sum of $\rho_{r}$, $\rho_{b}$, and $h_{b}$ is regarded as the social vulnerability factor, higher values of which indicate a higher social vulnerability that can trigger larger risk of social loss.

(4) $$IRE=\left|\bar{v}\right|\cdot \left|D\right|\cdot (\rho_{r}+\rho_{b}+h_{b})$$

(5) $$\rho_{r}=L_{r}/S_{w}$$

(6) $$\rho_{b}=S_{b}/S_{w}$$

(7) $${\bar{h}}_{b}=h_{b}\cdot \frac{S_{b}}{S_{w}}=h_{b}\cdot \rho_{b}$$

Specifically, in Equation (4), the mathematical sign || denotes the absolute values of the corresponding physical quantity. In Equation (5), with the retrieved dominant road network, the physical quantity of $\rho_{r}$ can be calculated by dividing the line length of traffic road networks $L_{r}$ with the acreage $S_{w}$ for each configured calculation window. In Equation (6), the spatial point density of local buildings $\rho_{b}$ can be retrieved with the ratio between the buildings’ spatial acreage $S_{b}$ and the acreage $S_{w}$ for each configured calculation window. In Equation (7), the average building height ${\bar{h}}_{b}$ can be calculated with the product between the buildings’ height $h_{b}$ and the spatial distribution density $\rho_{b}$ for each configured calculation window. In this study, the search window size is configured as the rectangle window with a size of 101 × 101 pixels for the calculation of $\rho_{r}$, $\rho_{b}$, and $h_{b}$. Additionally, based on the derived IRE values, the whole study region is evaluated by classifying the collapse risks into five levels of very low, low, medium, high, and extremely high.

## 3. Results

**Figure 3:**
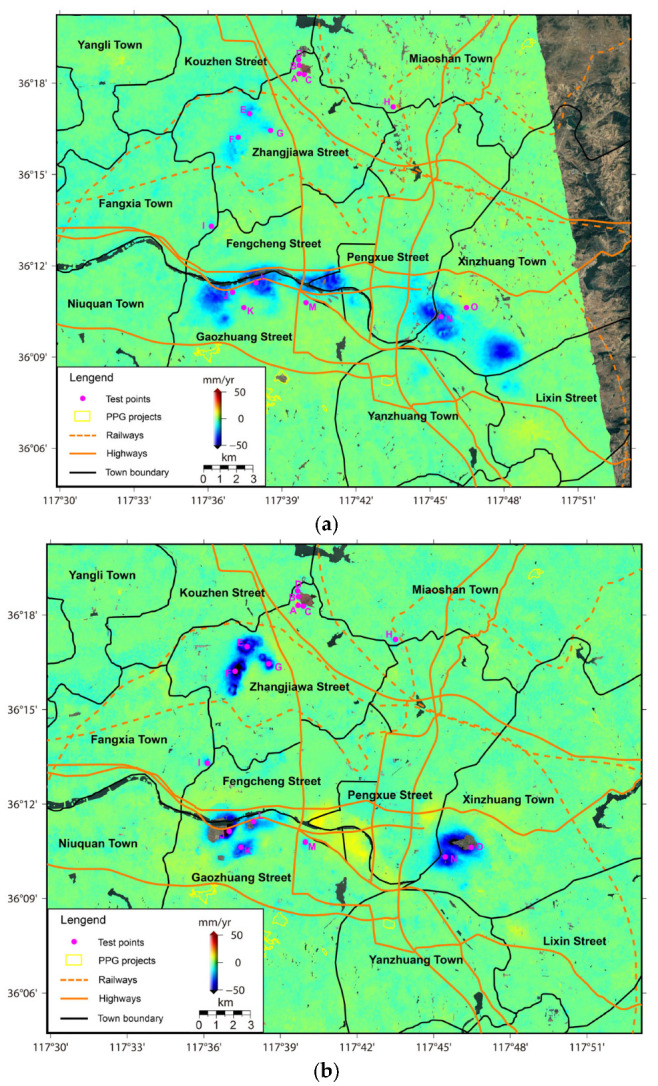
(**a**) ALOS PALSAR (17 January 2007~28 October 2010) and (**b**) Sentinel-1 (30 July 2015~22 August 2022) derived light-of-sight (LOS) deformation velocities (mm/year).

After performing the SBAS-InSAR processing chains on ALOS PALSAR and Sentinel-1 SAR SLC images, the ground deformation velocity fields in LOS directions of different observation sensors can be retrieved as presented in Figure 3a,b, respectively. The standard deviations (STDs) of deformation results in stable areas indicate a relatively low measurement uncertainty of approximately 1.38 mm/year for ALOS PALSAR, and approximately 1.39 mm/year for Sentinel-1. To better illuminate the deformation process, the historical ground deformation results of 15 test points (see Figure 1 and Figure 3) are compared with the environmental factors.

## 4. Discussion

### 4.1. Spatial Ground Deformation Evolution Characteristics

**Figure 4:**
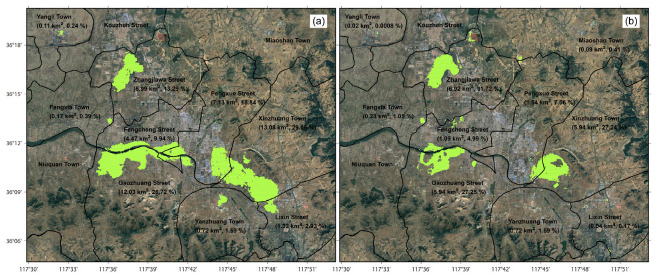
The identified LOS deformation region covered by green points for (**a**) ALOS PALSAR observations from 17 January 2007 to 28 October 2010, and (**b**) Sentinel-1 observations from 30 July 2015 to 22 August 2022.

With the filtering strategy of Section 2.2.2, ground deformation regions are outlined over different observation periods (see Figure 4), and quantitatively compared in view of administrative division as follows. Totally, as indicated in Figure 4a, the deformation region revealed by ALOS PALSAR (17 January 2007~28 October 2010) occupies around 45.03 km^2^ (−15.1 ± 8.0 mm/year), mainly distributed in Xinzhuang Town (13.08 km^2^, −16.0 ± 8.9 mm/year, 29.06%), Gaozhuang Street (12.03 km^2^, −16.8 ± 7.9 mm/year, 26.72%), Pengxue Street (7.13 km^2^, −16.4 ± 8.8 mm/year, 15.84%), Zhangjiawa Street (5.99 km^2^, −11.2 ± 3.4 mm/year, 13.29%), Fengcheng Street (4.47 km^2^, −14.7 ± 6.1 mm/year, 9.94%), Lixin Street (1.32 km^2^, −9.7 ± 2.6 mm/year, 2.93%), Yanzhuang Town (0.72 km^2^, −9.2 ± 1.8 mm/year, 1.59%), Fangxia Town (0.17 km^2^, −8.2 ± 1.0 mm/year, 0.39%), and Yangli Town (0.11 km^2^, −6.6 ± 1.3 mm/year, 0.24%). Comparatively, as indicated in Figure 4b, the deformation region revealed by Sentinel-1 (30 July 2015~22 August 2022) occupies around 21.81 km^2^ (−20.5 ± 12.3 mm/year), mainly distributed in Zhangjiawa Street (6.92 km^2^, −24.0 ± 13.7 mm/year, 31.72%), Gaozhuang Street (5.94 km^2^, −18.4 ± 11.0 mm/year, 27.25%), Xinzhuang Town (5.94 km^2^, −22.0 ± 11.9 mm/year, 27.24%), Pengxue Street (1.54 km^2^, −12.4 ± 6.3 mm/year, 7.06%), Fengcheng Street (1.09 km^2^, −15.2 ± 9.1 mm/year, 4.99%), Fangxia Town (0.23 km^2^, −15.3 ± 6.4 mm/year, 1.05%), Miaoshan Town (0.09 km^2^, −19.0 ± 6.3 mm/year, 0.41%), Lixin Street (0.04 km^2^, −10.0 ± 1.6 mm/year, 0.17%), and Yangli Town (0.02 km^2^, 9.9 ± 2.1 mm/year, 0.0008%).

Comparisons of ground deformation results over the observation periods of 17 January 2007~28 October 2010 (ALOS PALSAR) and 30 July 2015~22 August 2022 (Sentinel-1) show that the ground deformation regions are heavily concentrated in the Zhangjiawa-Yaojialing iron ore mining area (Maoci Village, Xiaowa Village, and Shantoudian Village), the Yujiaquan tailing ponds, Pocaowa-Dongwennan-Zhangjialou Villages, the urban area in Fengcheng Street, and the coal-mining region (Xinzhuang Town). However, relative to the ALOS PALSAR results, ground deformation regions revealed by Sentinel-1 display a distribution area reduced by around 51.57% but an averagely enhanced velocity of around −5.4 mm/year. Specifically, as displayed in Figure 4b, the deformation region near the Dawen River is gradually concentrated in Pocaowa-Dongwennan-Zhangjialou Villages. The original ground subsidence region in Fengcheng Street even presents slightly positive lifting signals. The spatial distribution of the ground deformation region in Laiwu coal fields is also decreased.

The dominant causes of a shrinking deformation area can be attributed to the remedial measures of local government performed over the observation period of Sentinel-1, including the reduction of groundwater extraction and ore-mining activities [12,13,14,15]. With Laiwu as an important transportation junction of central Shandong Province, several key traffic roads, including the highways, and the railways go through the ground deformation regions (see Figure 3), which should be further monitored by local administrations. Also, ground deformation regions are mostly located near to urban built-up areas including massive buildings and infrastructures. Hence, the density of road networks and the height of buildings are taken into consideration in risk evaluation.

**Figure 5:**
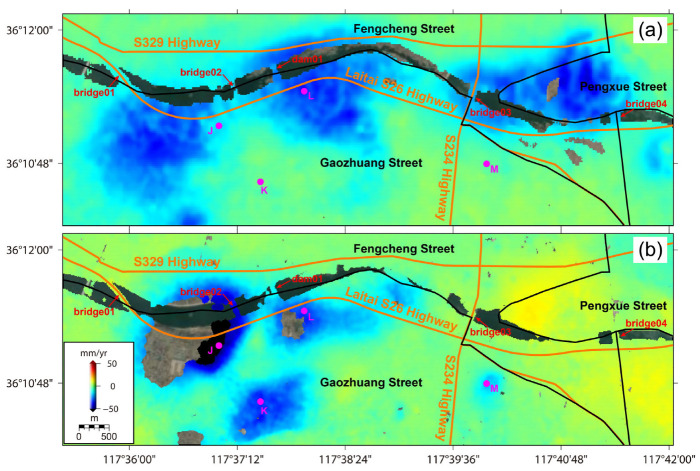
The LOS deformation velocities of the region near the banksides of the Dawen River derived from (**a**) ALOS PALSAR observations (17 January 2007~28 October 2010) and (**b**) Sentinel-1 observations (30 July 2015~22 August 2022). Notably, indicated by the magenta dots, 4 test points coded from J, K, L and M are located at the banksides of the Dawen River (J, K, L) and the coal mining region (M).

From the perspective of land types, as displayed in Figure 5a, many traffic road networks are distributed through the ground deformation region near the banksides of the Dawen River, including S329 Highway, Laitai S26 Highway, and S234 Highway. In addition, in the administrative region of Gaozhuang Street, Fengcheng Street, and Pengxue Street, many bridges and dams constructed over the Dawen River are confronted with different degrees of surface deformation. For example, bridge01, bridge02, bridge03, bridge04, and dam01 display dominant deformation in the range of −9~−25 mm/year, over the ALOS PALSAR observation period (17 January 2007~28 October 2010). However, as shown by the deformation velocity over the Sentinel-1 observation period (30 July 2015~22 August 2022) in Figure 5b, Laitai S26 Highway goes through two regions of serious ground subsidence. Moreover, bridge02 and dam01 are still confronted with ground subsidence risks, especially for bridge02 with the more serious deformation velocity in the range of −12~−42 mm/year. Additionally, the spots of high deformation velocity values observed on the right of Figure 5b demonstrate the difference triggered by different frequency for ALOS PALSAR and Sentinel-1 [29].

**Figure 6:**
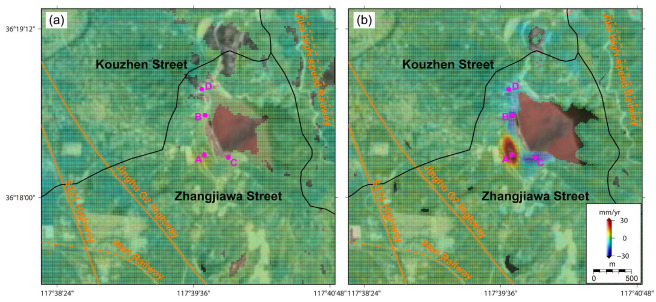
The LOS deformation velocities of the Yujiaquan tailing pond derived from (**a**) ALOS PALSAR observations (17 January 2007~28 October 2010) and (**b**) Sentinel-1 observations (30 July 2015~22 August 2022). Notably, indicated by the magenta dots, 4 test points coded from A, B, C and D are located at Yujiaquan tailing pond.

**Figure 7:**
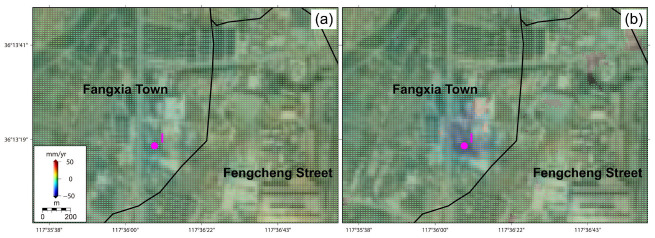
The LOS deformation velocities of the Yaojialing iron ore-mining region derived from (**a**) ALOS PALSAR observations (17 January 2007~28 October 2010) and (**b**) Sentinel-1 observations (30 July 2015~22 August 2022). Notably, the magenta point of I indicates the location of Yaojialing iron ore-mining region.

As displayed in Figure 6, the banksides of the Yujiaquan tailing pond display obvious deformation signals, posing a serious threat to the resident community (Huashuiquan Village, Linmazhuang Village, Beishanyang Village, Zhangjiazhuang Village, Fengjiazhuang Village, Nanshanyang Village, and Yujiaquan Village), the traffic roads (Jinghu G2 Highway, S234 Highway, Wari Railway, Jilai High-Speed Highway), and the industrial infrastructures (the Chuiyang iron concentrator) downstream. In detail, the main dam body of this tailing pond shows that the dominant positive deformation values reached approximately 32 mm/year, mainly triggered by the daily construction of this dam [18,19,20]. Comparatively, the flanks of the main dam body of this tailing pond indicate an obvious subsidence tendency with negative deformation values in range of −8~−26 mm/year. The collapse risks are, thus, mainly concentrated in the auxiliary dam in the northern orientation and the natural hillside in the southern orientation. Different from the obvious deformation patterns of the Yujiaquan tailing pond, as displayed in Figure 6 and Figure 7, the Yaojialing tailing pond and the Chuiyang tailing pond both display less of a deformation pattern, with magnitudes in range of 0.6~1.2 mm/year.

**Figure 8:**
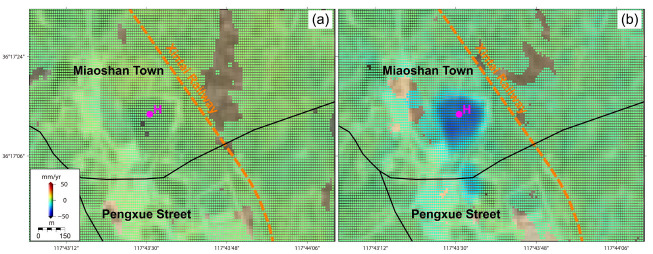
The LOS deformation velocities of the newly constructed landfill for industrial wastes derived from (**a**) ALOS PALSAR observations (17 January 2007~28 October 2010) and (**b**) Sentinel-1 observations (30 July 2015~22 August 2022). Notably, with the location indicated by the magenta point of H, this landfill was constructed from July 2019 to December 2019.

Moreover, as displayed in Figure 8, serious ground subsidence signals of approximately −28 mm/year are also found in a newly constructed landfill for industrial waste material, possibly triggered by the excavation of this industrial infrastructure [30]. The spatial and temporal evolution patterns of the ground deformation in this landfill are largely consistent with the spatial range and temporal period of the corresponding construction process. Additionally, some photovoltaic power generation (PPG) infrastructures are also distributed in the original mountainous coal-mining region of the study area. However, different from the active deformation state of PPG in Xintai City [28], these PPG infrastructures display lower ground deformation signals and maintain a relatively stable operating state, with magnitudes in the range of 0.5~1.3 mm/year (see Figure 3).

### 4.2. Temporal Ground Deformation Evolution Characteristics and Potential Triggering Mechanism

**Figure 9:**
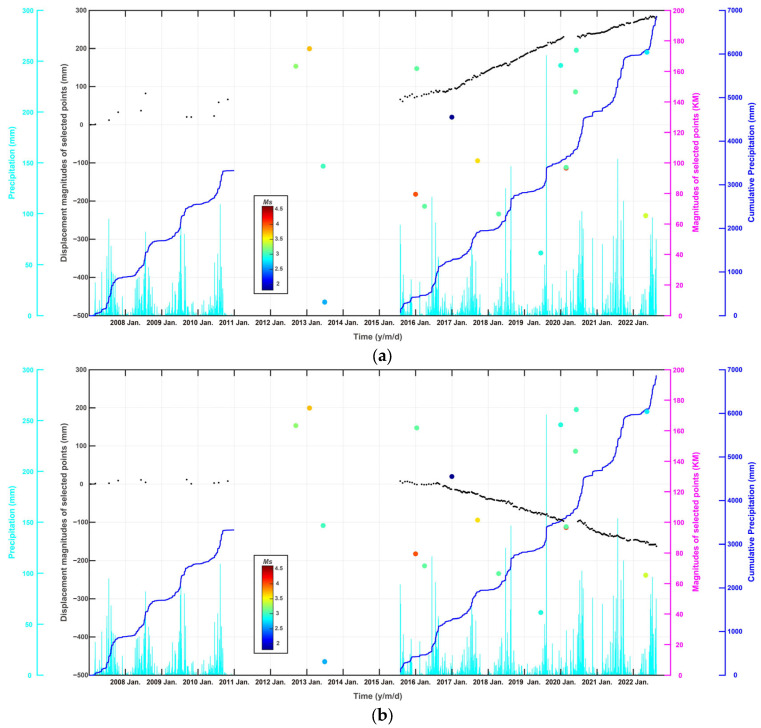

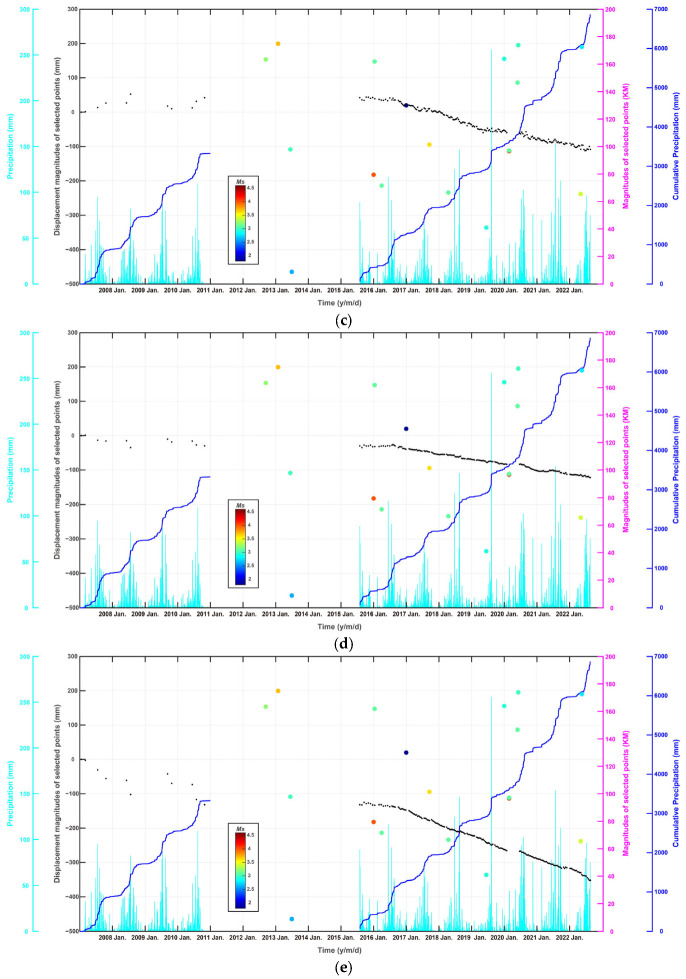

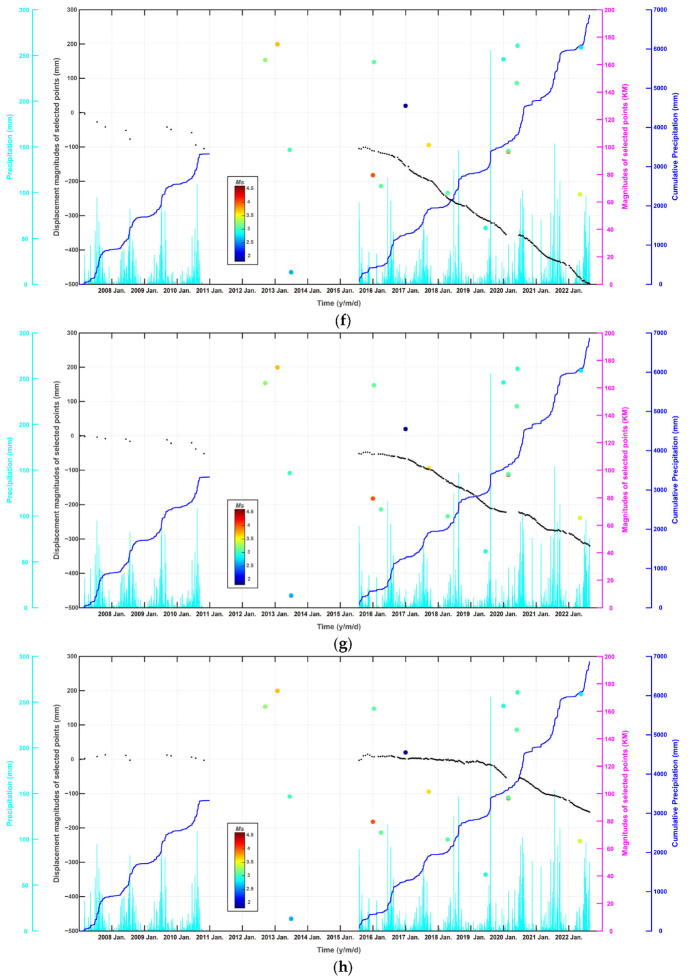

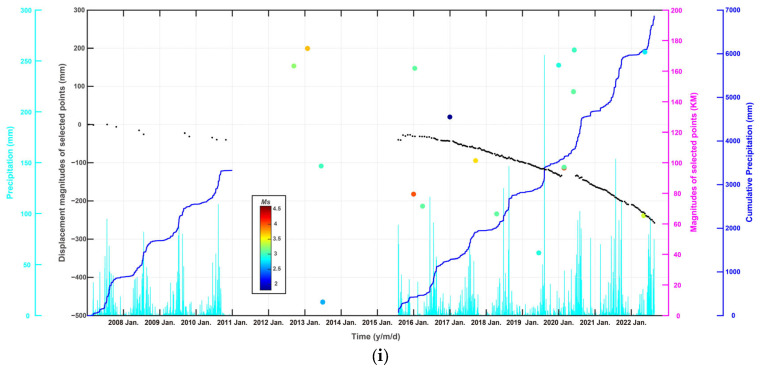
The time-series LOS deformation for test points of (**a**–**i**), in which A, B, C, and D are located on the banksides of the Yujiaquan tailing pond; E, F, and G are located in the traditional Luzhong iron ore-mining region; H is located in a newly constructed landfill for industrial wastes; I is located in the Yaojialing iron ore-mining region. In addition, the LOS deformation time series of J, K, L, M, N, and O can be found in Figures S1–S6. The environmental factors incorporating the daily precipitation, the cumulative precipitation, and the earthquake events, are cross-compared to the deformation time series derived from ALOS PALSAR and Sentinel-1 SAR observations.

To better illuminate the ground deformation process and the potential triggering factors, 15 test points (see Figure 1) are selected and displayed in Figure 1 and Figure 3, with four points (A, B, C, D) in the Yujiaquan tailing pond, one point (H) in the landfill, four points (E, F, G, I) in the Luzhong iron ore-mining area, three points (J, K, L) near the banksides of the Dawen River, and three points (M, N, O) in the traditional coalfield. Many studies have demonstrated that the environmental factors possibly controlling the ground deformation are mainly the precipitation and local earth dynamic events [31]. As shown in Figure 9 and Figures S1–S6, ground deformation time series curves of these 15 test points are, thus, further analyzed by comparison with local precipitation and earthquake events.

For the Yujiaquan tailing pond, four test points of A, B, C, and D are selected to derive the deformation evolution characteristics. As displayed in Figure 9a, due to the daily construction of the dam bank, point A of the tailing pond demonstrates a linearly increasing tendency with a total cumulative deformation reached at around 300 mm. However, as displayed in Figure 9b–d, the points B, C, and D of the tailing pond demonstrate a linear subsidence tendency, with the cumulative subsidence magnitudes reached at around 200 mm, 150 mm, and 100 mm, respectively. Different from the well-known, common internal mechanism of ground subsidence, ground deformation curves of these test points (see Figure 9a,b) still indicate a linear evolution tendency with the maximal summer precipitation of approximately 200 mm/day. Similarly, ground deformation curves also indicate a stable linear evolution tendency with a maximal earthquake magnitude of approximately 4.2 having occurred nearby.

As displayed in Figure 9e–g, the three test points of E, F, and G in the Luzhong iron ore-mining region demonstrate the largest deformation magnitudes of the whole study region, with final cumulative deformation magnitudes reached at approximately 300 mm, 500 mm, and 300 mm, respectively. However, these deformation curve time series generally entered into a quiescent state with deformation velocities close to zero in rainy months, and entered into the active state with deformation velocities largely increased in arid months. With the well-developed karst groundwater system, the internal dynamic mechanism is largely controlled by the seasonal changes in underground water conditions [9,10,11]. In rainy months, when the underground karst caves are filled with Quaternary pore water supplied by rainwater [32], the increased lifting force of the karst groundwater resists gravity coming from the ground surface and facilitates the decrease in, and even quiescence of, the ground deformation process. When the underground water level in the karst caves decreases, the lifting force of underground karst water is weakened and gradually become smaller than the gravity coming from ground surface, facilitating a large acceleration of the ground deformation process. As displayed in Figure 9h, the ground deformation curve of H in the newly constructed landfill demonstrates that the occurrence of a large deformation subsidence reached approximately 150 mm after July 2019, consistent with the construction period of this landfill.

Comparatively, as displayed in Figure 9i, the deformation time series curve of I in the Yaojialing iron ore-mining region indicates a dominant exponential-shaped increasing tendency, less affected by the external environmental factors of the precipitation and local earthquake events. This exponential-shaped curve pattern means a rapidly increasing deformation velocity, commonly regarded as an early warning of ground collapse [31]. The deformation time series of I and its nearby mining area, thus, have entered into the accelerating deformation stage and may possibly evolve into a ground collapse event in future. Considering the small distance of only 6 km between I and the urban center, local safety departments are suggesting checks as to whether the mining operation of the Yaojialing mine conforms to the safety operation standards or not.

As displayed in Figures S1–S3, the three test points of J (farmland), K (folk house), and L (farmland) on the banksides of the Dawen River were monitored and cumulative magnitudes reached 400 mm, 250 mm, and 350 mm, respectively. The deformation curve time series of J in the Jielin coal mine indicates a long-term linear deformation stage and a short-term quiescence stage. Comparatively, the deformation curve time series of L demonstrates a linear deformation stage, an exponential-shaped accelerated deformation stage, and a short-term quiescence stage. Specifically, the exponential-shaped accelerated deformation stage of L starts after the occurrence of the strongest precipitation in summer. Limited by the extraction of underground water, the evolution of underground water conditions is the dominant triggering force controlling the deformation pattern of L. However, the deformation curve time series of K in Pocaowa Village shows a similar exponential-shaped decreasing curve with the deformation velocity gradually decreasing to zero.

As displayed in Figure S4, the ground deformation time series of M in the coal mine region of Ezhuang Village demonstrates a dominant deformation subsidence tendency over the temporal periods of June 2015~June 2016 and May 2019~May 2021. Two test points of N and O are also selected in the traditional Xinzhuang coal-mining region to reveal the ground deformation process. As displayed in Figure S5, the ground deformation time series of N indicates a similar exponential-shaped decreasing curve, with the deformation velocity gradually decreasing to zero. Comparatively, as displayed in Figure S6, the ground deformation time series of O indicates a similar exponential-shaped increasing curve with the deformation velocity gradually increasing. However, some seasonal fluctuations are also observed in the ground deformation time series of O. Different deformation patterns of these two test points are possibly triggered by different engineering stages of local coal mining operations [28,32,33]. Specifically, the test point of N is located in a coal-mining region in a late stage of its coal-mining operation with moderate deformation subsidence. In contrast, the test point of O is located in a coal-mining region in the development stage of its coal mining operation with gradually increasing deformation subsidence.

### 4.3. Evaluation of the Potential Ground Collapse Risks

**Figure 10:**
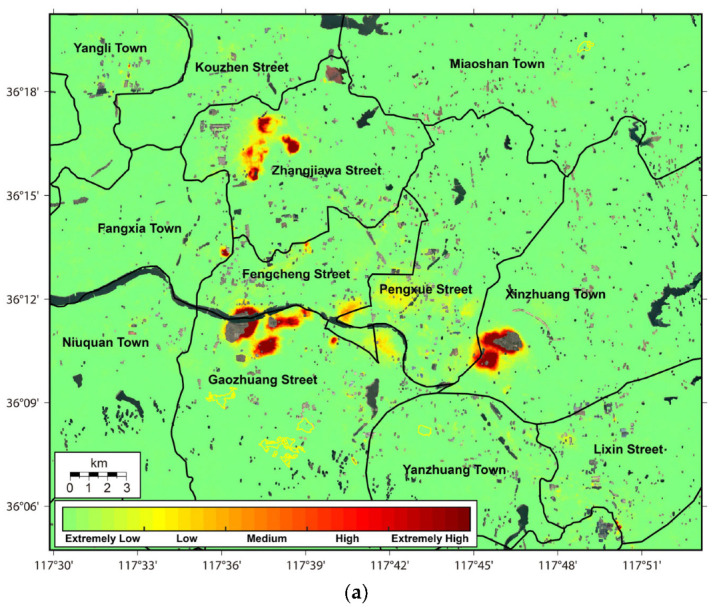

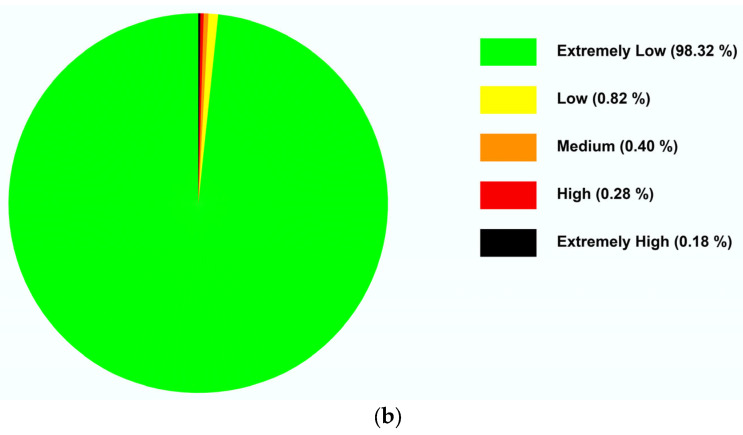
(**a**) The risk level map and (**b**) corresponding statistical pie chart of the traditional steel production base in Laiwu.

The whole study region is evaluated with the methodology in Section 2.2.2 as the five risk levels of extremely low, low, medium, high, and extremely high. As displayed in Figure 10, the percentages of these risk levels are 98.32% (942.30 km^2^), 0.82% (7.83 km^2^), 0.40% (3.82 km^2^), 0.28% (2.65 km^2^), and 0.18% (1.72 km^2^), respectively. Most regions with potential ground collapse risks indicate a high spatial consistency with industrial infrastructures and ore-mining regions constructed for local steel production. In detail, with a total area of around 8.19 km^2^, the potential collapse risks at the levels of medium, high, and extremely high are mainly distributed in Gaozhuang Street (2.71 km^2^, 33.11%), Xinzhuang Town (2.48 km^2^, 30.25%), Zhangjiawa Street (1.88 km^2^, 22.94%), Fengcheng Street (0.59 km^2^, 7.15%), Pengxue Street (0.34 km^2^, 4.12%), Fangxia Town (0.13 km^2^, 1.57%), Lixin Town (0.05 km^2^, 0.60%), Yangli Town (0.01 km^2^, 0.14%), Aishan Town (0.01 km^2^, 0.11%), and Yanzhuang Town (0.0012 km^2^, 0.01%). As aforementioned in Section 4.2, ground collapse risks are mainly triggered by massive underground mining activities and the sporadic engineering of industrial infrastructures. Some emergent infrastructures demonstrate an effectiveness for ecological remediation, such as PPG projects with a collapse risk of extremely low. However, different from other risk evaluation methods [34,35,36], the classifications of risk levels designed in this study are not intelligent enough. More artificial intelligence algorithms, such as the random forest-tree [37] and the convolutional neural network [38], will be considered in future work.

## 5. Conclusions

In this study, using the SBAS-InSAR technique with multi-temporal ALOS PALSAR and Sentinel-1 SAR observations, ground deformations of the traditional steel production base in Laiwu are derived to better investigate the internal triggering mechanism and evaluate potential collapse risks. Ground deformation signals are mainly concentrated in the traditional iron and coal ore-mining region, the banksides of the Dawen River, and some industrial infrastructures. Moreover, comparison between ALOS PALSAR and Sentinel-1 observations indicates that the deformation area dominantly presents a shrinking tendency as time goes by. Based on the time-series analysis on the ground deformation process of 15 test points, seasonal deformation patterns of a traditional iron and coal ore-mining region are dominantly affected by precipitation, underground water levels, and human activities.

To better evaluate the potential collapse risks, some important industrial infrastructures are highlighted. For example, the Yujiaquan tailing pond shows a linear lifting deformation pattern in the main dam bank that reached 300 mm and a linear subsidence deformation pattern in the auxiliary dam bank that reached 100 mm. Also, the test point in the Yaojialing iron mine shows an exponential-shaped increasing deformation curve, meaning a potential collapse risk of the ground surface. Furthermore, through constructing an IRE, the whole study region is evaluated with five risk levels of extremely low (98.32%), low (0.82%), medium (0.40%), high (0.28%), and extremely high (0.18%). This comprehensive remote sensing monitoring result can better serve the economic and social development of these resource-based industrial cities.

## Figures and Tables

**Table 1:** Acquisition details of Sentinel-1 and ALOS PALSAR sensors.

Sensors	Sentinel-1	ALOS PALSAR
Operating Time	12 April 2014~Now	24 January 2006~22 April 2011
Frame/Path	111/142	710/447
Heading Direction	Ascending	Ascending
Incidence Angle	20°~46°	18°~70°
Bands	C	L
Polarization	VV	HH
Number of Images	176	22
Acquisition Time	30 July 2015~22 August 2022	17 January 2007~28 October 2010

## Data Availability

Data are contained within the article.

## Supplementary Materials

The following supporting information can be downloaded at: https://www.mdpi.com/article/10.3390/s24154872/s1, Figure S1: The LOS deformation time series of J; Figure S2: The LOS deformation time series of K; Figure S3: The LOS deformation time series of L; Figure S4: The LOS deformation time series of M; Figure S5: The LOS deformation time series of N; Figure S6: The LOS deformation time series of O.
