# Supplementary material for: Retrospect on the Ground Deformation Process and Potential Triggering Mechanism of the Traditional Steel Production Base in Laiwu with ALOS PALSAR and Sentinel-1 SAR Sensors
Source: Sensors (Basel). 2024 Jul 26;24(15):4872. doi: 10.3390/s24154872 (PMC11315039; doi:10.3390/s24154872)
Supplement: Supplementary file 1 [file sensors-24-04872-s001.zip › sensors-3062919-supplementary.pdf]

Supplementary material for

Retrospect on the ground deformation process and potential triggering mechanism of traditional steel production base in Laiwu with ALOS PALSAR and Sentinel-1 SAR sensors

Chao Ding[a,c,*], Guangcai Feng[b], Lu Zhang[c], Wenxin Wang[b]

[a] *School of Civil Engineering and Geomatics, Shandong University of Technology, Zibo 255049, China;*

[b] *School of Geosciences and Info-Physics, Central South University, Changsha 410083, Hunan, China;*

[c] *State Key Laboratory of Information Engineering in Surveying, Mapping and Remote Sensing, Wuhan University, Wuhan 430079, Hubei, China;*

***Correspondence author:** *Chao Ding, E-mail address: dingchao_whu@whu.edu.cn*

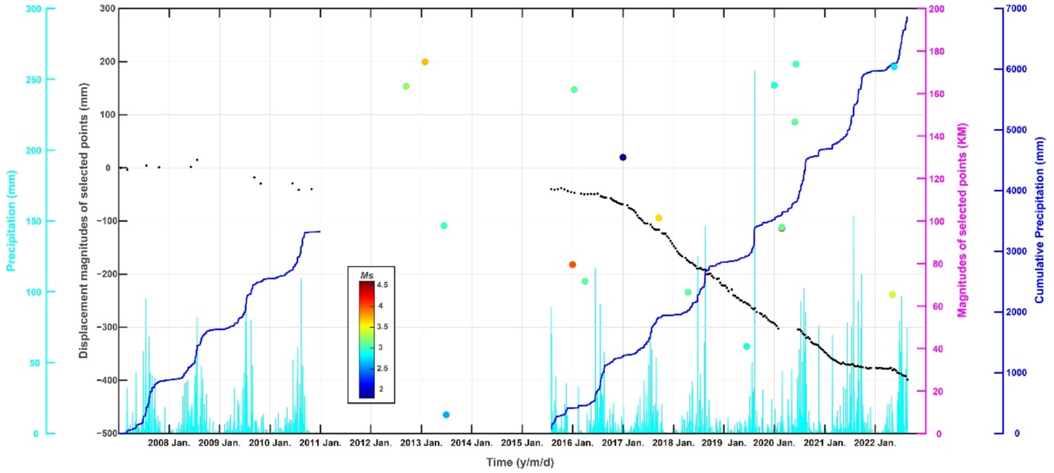

Figure S1. The LOS deformation time series of J located in the banksides of Dawen River. The environmental factors incorporating the daily precipitation, the cumulative precipitation, and the earthquake events, are cross-compared to the ground deformation time series derived from ALOS PALSAR and Sentinel-1 SAR observations.

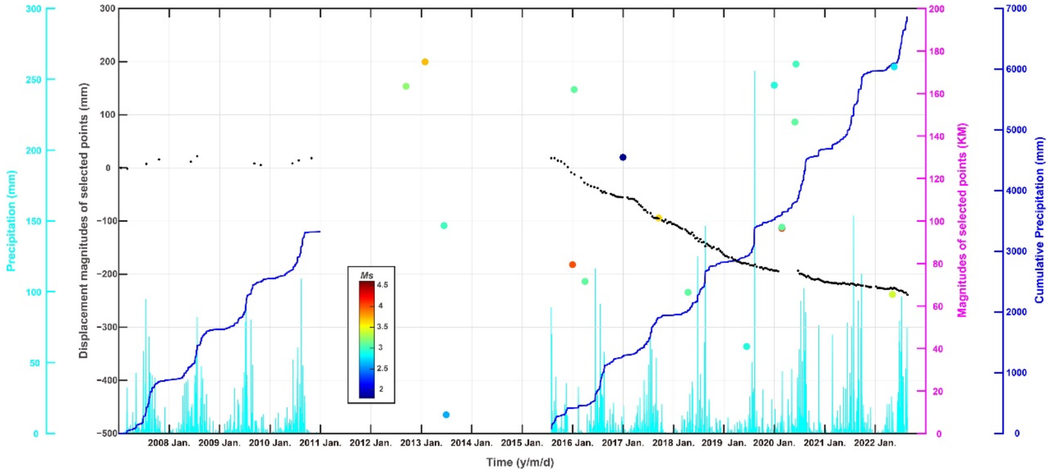

Figure S2. The LOS deformation time series of K located in the banksides of Dawen River. The environmental factors incorporating the daily precipitation, the cumulative precipitation, and the earthquake events, are cross-compared to the ground deformation time series derived from ALOS PALSAR and Sentinel-1 SAR observations.

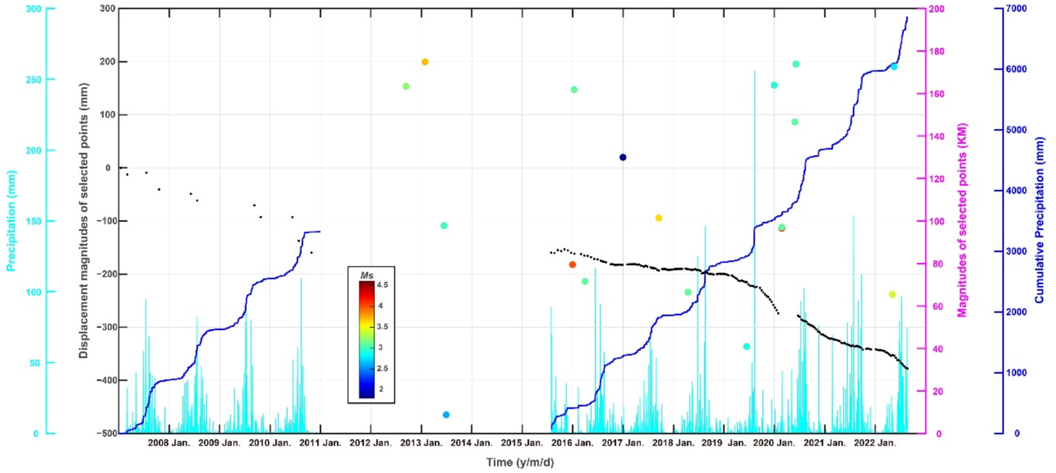

Figure S3. The LOS deformation time series of L located in the banksides of Dawen River. The environmental factors incorporating the daily precipitation, the cumulative precipitation, and the earthquake events, are cross-compared to the ground deformation time series derived from ALOS PALSAR and Sentinel-1 SAR observations.

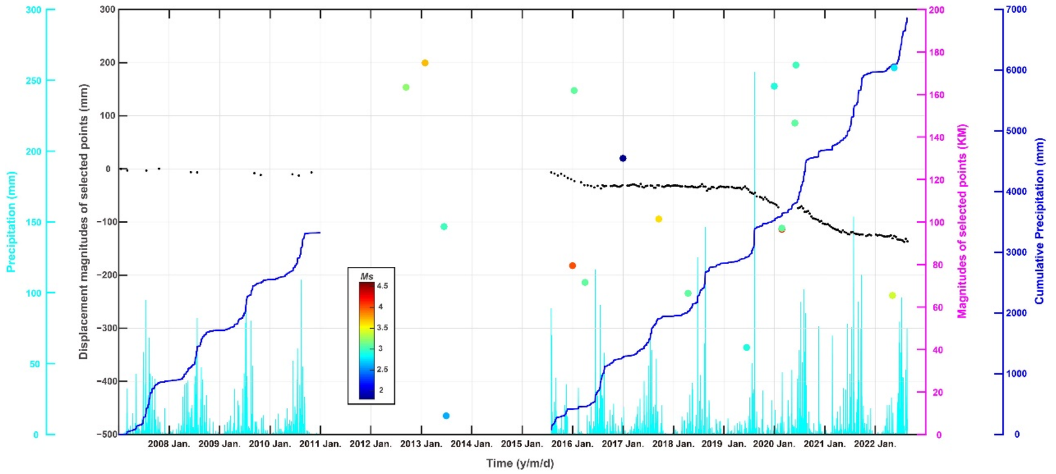

Figure S4. The LOS deformation time series of M located in the traditional Laiwu coalfields. The environmental factors incorporating the daily precipitation, the cumulative precipitation, and the earthquake events, are cross-compared to the ground deformation time series derived from ALOS PALSAR and Sentinel-1 SAR observations.

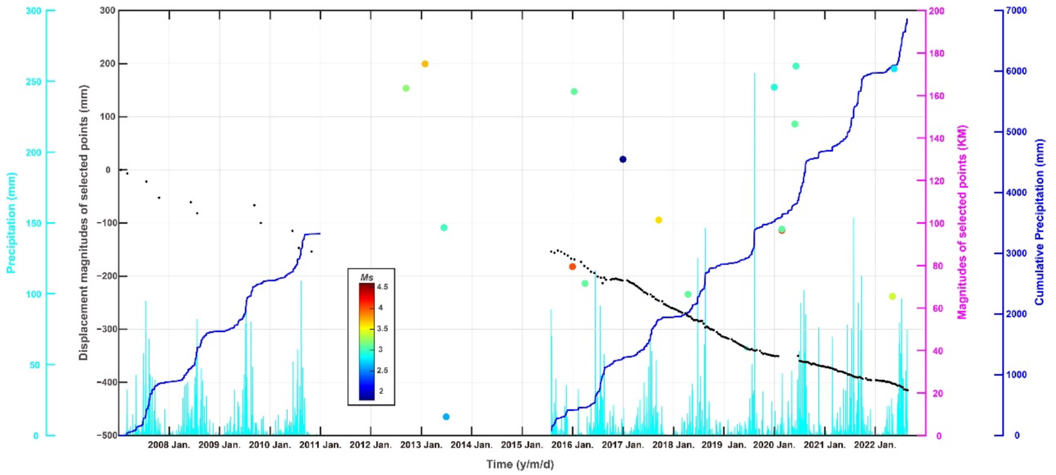

Figure S5. The LOS deformation time series of N located in the traditional Laiwu coalfields. The environmental factors incorporating the daily precipitation, the cumulative precipitation, and the earthquake events, are cross-compared to the ground deformation time series derived from ALOS PALSAR and Sentinel-1 SAR observations.

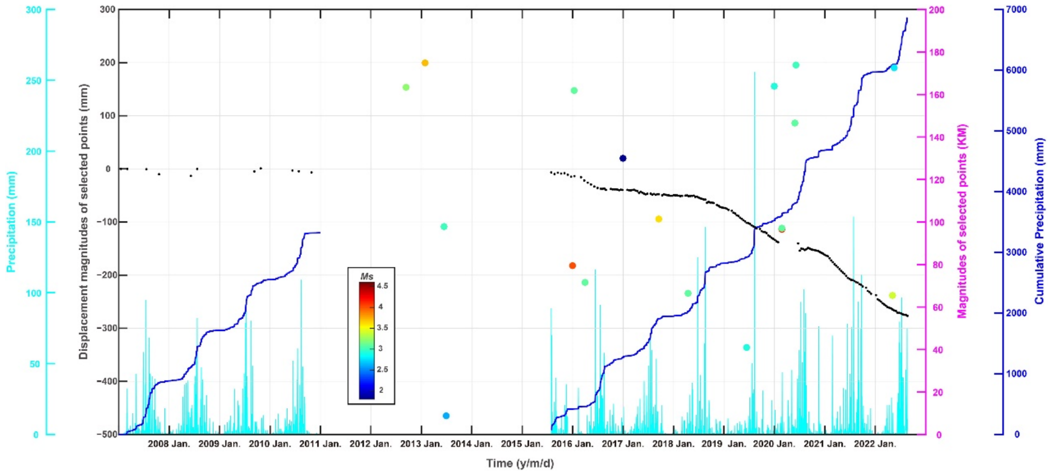

Figure S6. The LOS deformation time series of O located in the traditional Laiwu coalfields. The environmental factors incorporating the daily precipitation, the cumulative precipitation, and the earthquake events, are cross-compared to the ground deformation time series derived from ALOS PALSAR and Sentinel-1 SAR observations.
